# Placental Insulin Receptor Transiently Regulates Glucose Homeostasis in the Adult Mouse Offspring of Multiparous Dams

**DOI:** 10.3390/biomedicines10030575

**Published:** 2022-03-01

**Authors:** Grace Chung, Ramkumar Mohan, Megan Beetch, Seokwon Jo, Emilyn Uy Alejandro

**Affiliations:** Department of Integrative Biology and Physiology, University of Minnesota Medical School, University of Minnesota, Minneapolis, MN 55455, USA; chung491@umn.edu (G.C.); rammohan@med.umich.edu (R.M.); beet0013@umn.edu (M.B.); joxxx057@umn.edu (S.J.)

**Keywords:** placental insulin receptor, gestational diabetes, fetal programming, multiparity, obesity, type 2 diabetes

## Abstract

In pregnancies complicated by maternal obesity and gestational diabetes mellitus, there is strong evidence to suggest that the insulin signaling pathway in the placenta may be impaired. This may have potential effects on the programming of the metabolic health in the offspring; however, a direct link between the placental insulin signaling pathway and the offspring health remains unknown. Here, we aimed to understand whether specific placental loss of the insulin receptor (InsR) has a lasting effect on the offspring health in mice. Obesity and glucose homeostasis were assessed in the adult mouse offspring on a normal chow diet (NCD) followed by a high-fat diet (HFD) challenge. Compared to their littermate controls, InsR KO^placenta^ offspring were born with normal body weight and pancreatic β-cell mass. Adult InsR KO^placenta^ mice exhibited normal glucose homeostasis on an NCD. Interestingly, under a HFD challenge, adult male InsR KO^placenta^ offspring demonstrated lower body weight and a mildly improved glucose homeostasis associated with parity. Together, our data show that placenta-specific insulin receptor deletion does not adversely affect offspring glucose homeostasis during adulthood. Rather, there may potentially be a mild and transient protective effect in the mouse offspring of multiparous dams under the condition of a diet-induced obesogenic challenge.

## 1. Introduction

The growing prevalence of obesity and diabetes in pregnant women and women of reproductive age have become major concerns in women’s health, with over 60% of women of reproductive age being obese or overweight and an increasing number of pregnancies complicated by gestational diabetes mellitus (GDM) [1,2,3,4]. Maternal obesity and diabetes during pregnancy are associated with short- and long-term adverse pregnancy complications for both the mother and the baby [5,6,7,8,9,10]. Exposure to maternal obesity or diabetes in utero is associated with an increased risk for child and adult obesity, type 2 diabetes (T2D), cardiovascular disease, and neurodevelopmental disorders in the offspring [7,11,12,13,14,15]. Thus, maternal obesity and diabetes during pregnancy are important considerations in the programming of the metabolic health in the offspring and may have intergenerational effects, further perpetuating the vicious cycle of obesity and diabetes [9,16]. An important risk factor of maternal obesity is multiparity, which exacerbates gestational weight gain, inflammation, and risk of adverse metabolic outcomes in the offspring [17,18,19].

The placenta is critical for the development and growth of the fetus, and evidence suggests that the placenta plays an important role in the long-term health of the offspring [20,21,22,23,24,25]. Insulin is an important growth factor and regulates placental and fetal growth, nutrient transfer, and hormone secretion [26,27,28]. In early pregnancy, maternal insulin response has been associated with placental weight at birth, which has been associated with neonatal birth weight and adiposity at term [27]. In pregnancies complicated by maternal obesity and GDM, there is evidence to suggest that the insulin signaling pathway in the placenta may be impaired and may contribute to changes in the metabolic programming of the offspring [26,28,29,30,31,32]. However, a direct link between the placental insulin signaling pathway and the metabolic health of the offspring remains unknown. 

Despite strict glycemic control in the modern clinical management of pregnant women with prediabetes and GDM, fetal overgrowth remains an important clinical problem [33]. Further, insulin therapy during pregnancy is potentially associated with a risk of developing T2D in the offspring later in life, but it has not been investigated directly. Jansson et al. highlights the importance of placental function and the possible role of maternal insulin [34]. Therefore, a greater understanding of the programming impact of maternal insulin on the metabolic health of the offspring will be significant in illuminating the effects of insulin therapy on the children of women with GDM, T1D, or T2D during pregnancy. 

Due to the limitations of human clinical studies (i.e., ethical issues), preclinical models lend feasibility to assess how specific features of maternal health such as hyperinsulinemia contribute to the programming of the metabolic health in the offspring. Therefore, there is a need for preclinical studies to specifically assess the role of the placental insulin receptor in the metabolic health trajectory of the adult offspring.

The placental insulin receptor (InsR) impacts the nutrient flux from the mother to the fetus and may affect the developing insulin-producing β-cells, which are highly sensitive to changes in the nutrient flux in utero [35]. Moreover, the roles for maternal insulin and placental InsR in the metabolic health of the offspring are untested, and we have the state-of-the art preclinical model to address this gap in knowledge. Therefore, in the present study, we aimed to understand whether specific placental loss of the insulin receptor has a lasting effect on the fetus, altering the birthweight and the long-term metabolic health trajectory of the mouse offspring. Mice with a genetic specific deletion of the insulin receptor in the placental trophoblast (Cyp19-cre; InsR^f/f^ hereinafter, referred to as InsR KO^placenta^) were generated using the cre/loxP system described further in the Methods section [23,36,37,38]. Fetal and newborn body weight and pancreatic β-cell mass were assessed in littermate male and female offspring. Obesity and glucose homeostasis were assessed in the adult offspring from multiparous and non-multiparous dams on a normal chow diet (NCD), followed by a high-fat diet (HFD) challenge. 

## 2. Materials and Methods

### 2.1. Generation of the Animal Mouse Model and Diet

The cre/loxP system is a novel and powerful tool to generate mice with a tissue-specific knockout of a specific gene. Under the control of the human *Cyp19* promoter, Cyp19-cre is a placenta-specific cre recombinase active in the mouse spongiotrophoblast and syncytiotrophoblast cells [38]. Cyp19-cre mice were generated and gifted by Dr. Gustavo Leone (Medical College of Wisconsin) [38]. In this present study, conditional deletion of the LoxP-flanked InsR gene in the placental trophoblast cells may allow for better understanding of how the insulin signaling pathway can affect placental and fetal development. To generate placental trophoblast-specific insulin receptor knockout mice (Cyp19-cre; InsR^f/f^) and their littermate controls (InsR^f/f^), floxed InsR male mice (InsR^f/f^) (purchased from Jackson Laboratory, stock No. 006955) were bred with floxed InsR female mice expressing the placenta-specific Cre recombinase (Cyp19-cre; InsR^f/f^). For mating, one male was placed in a cage with two females. The male was then separated while the dams were pregnant. For multiparous females, pups were born and allowed to suckle. The pups were weaned at 21–28 days postpartum, and the females were remated within 5 days of weaning, and the process repeated. The dams were allowed to give birth to up to four litters. 

The placenta and the associated extraembryonic membranes are formed from the zygote at the start of each pregnancy and thus have the same genetic composition as the fetus. Therefore, the placental genotypes were determined by the offspring genotypes (whether they are control or KO), and this was done prior to weaning using standard PCR on the tissue collected on postnatal (P) day 6–8. The primers used are listed below: CYP19cre forward: GACCTTGCTGAGATTAGATC; CYP19cre reverse: GACGATGAAGCATGTTTAGCT GGCC; InsR tm1Khn forward: GGG GCA GTG AGT ATT TTG GA; and InsR tm1Khn reverse: TGG CCGTGA AAGTTAAGAGG. Validation of the efficiency of Cre was assessed by our group and others [23,37]. All the mice were group-housed under a 14:10 light–dark cycle with ad libitum access to food. At 13 weeks of age, the mice were switched to a high-fat rodent diet with 60 kcal% fat (Research Diets, Inc., D12492) until the time of harvest. The adult offspring under a normal chow diet and a high-fat diet were from 2–3 dams. All the animal studies were performed in accordance with the University of Minnesota Institutional Animal Care and Use Committee (protocol #2106-39213A).

### 2.2. Newborn Pancreas Collection

The pregnant dams were allowed to deliver spontaneously, with the morning of the vaginal plug denoting E0.5. The newborns were separated out from the cage and euthanized by decapitation. Blood was collected and centrifuged at 10,000× *g* for 10 min at room temperature. The supernatant serum was collected and stored at −80 °C to be run for serum insulin concentration using an ALPCO ELISA kit as per the kit’s instructions (ALPCO Mouse Ultrasensitive Insulin ELISA; ALPCO Rat High Range Insulin ELISA, ALPCO Diagnostic, Salem, NH, USA). A five-parameter logistic fit was used for analysis through the MyAssays software. Newborn pancreata were harvested, weighed, fixed in 3.7% formalin for 4–6 h, and stored in 70% EtOH (diluted with 1× PBS) at 4 °C until tissue processing. The tissues were processed under the normal tissue processing settings and embedded in paraffin. The paraffin-embedded pancreata were sectioned 5 microns apart from top to bottom. Tails from the newborn mice were collected separately during harvesting for genotyping by standard PCR (see the information about the primers above). The pups for embryonic data were collected from three dams. The newborn data were collected from six dams. 

### 2.3. Glucose Tolerance Test

The mice were fasted overnight for 14 h, after which fasting body weight and fasting blood glucose were measured. A dosage of 2 g/kg of 50% dextrose solution (Hospira, Inc., Lake Forest, IL, USA) was administered intraperitoneally. Blood glucose levels were recorded through a small tail clip at 30, 60, and 120 min after the initial injection. Blood glucose was measured using a Bayer Contour Blood Glucose Monitoring System. The adult offspring under a normal chow diet and a high-fat diet were from 2–3 dams.

### 2.4. Insulin Tolerance Test

The mice were fasted for 6 h, beginning in the morning, after which fasted body weight and fasted blood glucose were measured. A dosage of 0.75 U/kg insulin (Humalog, Eli Lilly, Indianapolis, IN, USA) in 0.9% sterile saline was administered intraperitoneally, and blood glucose levels were recorded through a small tail clip at 30, 60, and 120 min after the initial injection. Blood glucose was measured using a Bayer Contour Blood Glucose Monitoring System. The data are presented as the baseline percentage (%) corrected to the blood glucose level taken at T = 0 min after a 6 h fast.

### 2.5. Body Composition and Indirect Calorimetry

Body composition (EchoMRI, Echo Medical Systems LLC, Houston, TX, USA) and indirect calorimetry (Oxymax/CLAMS Lab Animal Monitoring System, Columbus Instruments) were performed by the Integrative Biology and Physiology (IBP) Core at the University of Minnesota. 

### 2.6. Immunofluorescence and H&E Staining

For immunofluorescence imaging, paraffin-embedded tissue sections were deparaffinized and rehydrated, followed by antigen retrieval in citric buffer by boiling. The sections were incubated with primary antibodies against guinea pig or mouse insulin (guinea pig insulin primary antibody (1:400; DAKO, Agilent, Santa Clara, CA, USA); mouse insulin primary antibody (1:400; Abcam, Waltham, MA, USA)) at 4 °C overnight. The sections were subsequently washed with 1× PBS + 0.01% Tween 20 (1× PBST) with mild shaking and incubated with secondary antibodies conjugated to FITC (fluorescein isothiocyanate, Jackson ImmunoResearch, West Grove, PA, USA), a bright green fluorophore, for 1.5 h at 37 °C. Following incubation, the sections were subsequently washed with 1× PBST and counterstained for nuclei using a DAPI (4′,6-diamidino-2-phenylindole, Fisher Scientific, Hampton, NH) dip, a blue fluorescent stain with a high affinity for DNA. The sections were then cover-slipped with a mounting media and imaged on a motorized microscope (Nikon Eclipse NI-E; Nikon, Melville, NY, USA). H&E was used to assess macroscopic observation of the mouse placenta. Sagittal cuts were performed on the middle section of mouse placenta paraffin-embedded cut side down. The standard Citri Solv (Thermo Fisher Scientific, Waltham, MA, USA) deparaffinization and dehydration procedure was performed on the mouse placenta tissue. H&E staining was performed as per the manufacturer’s protocol. Placental tissues were visualized using a Nikon ECLIPSE NI-E microscope. 

### 2.7. Beta Cell Mass Analysis

The newborn paraffin-embedded pancreata were sectioned 5 microns apart from top to bottom per animal. The sections stained for insulin were selected incrementally at 100 μM apart, covering the whole pancreas. The sections were incubated with the insulin primary antibody overnight at 4 °C (see the dilution information above). The area of insulin-positive cells divided by the total pancreas area was quantified using FIJI ImageJ (NIH, Bethesda, MD, USA) to give the β-cell area/total pancreas area ratio per animal. The ratio was then multiplied by the matching pancreas weight (β-cell area/pancreatic area × pancreas weight) and averaged to give an average β-cell mass, as previously described [39]. The stained newborn tissue sections were imaged at 10× magnification using a Nikon Eclipse NI-E (Nikon Instruments) microscope. Individual islets were imaged at 20× magnification.

### 2.8. Statistical Analysis

All the reported values are expressed as the mean ± standard error of mean (SEM). Analyses of repeated data measures were performed using repeated measures two-way ANOVA with tests for sphericity. Individual offspring were the repeated measures subjects without regard to dams. An unpaired *t*-test was performed to analyze data where only two groups were compared. The area under the curve (AUC) values were calculated with zero as the baseline value. All the statistical analyses were completed using GraphPad Prism (San Diego, CA, USA) version 8 with a significance threshold of *p* ≤ 0.05.

## 3. Results

### 3.1. Newborn Offspring with Placenta-Specific InsR Ablation Presenting with Normal Body Weight and Pancreatic β-Cell Mass

**Figure 1:**
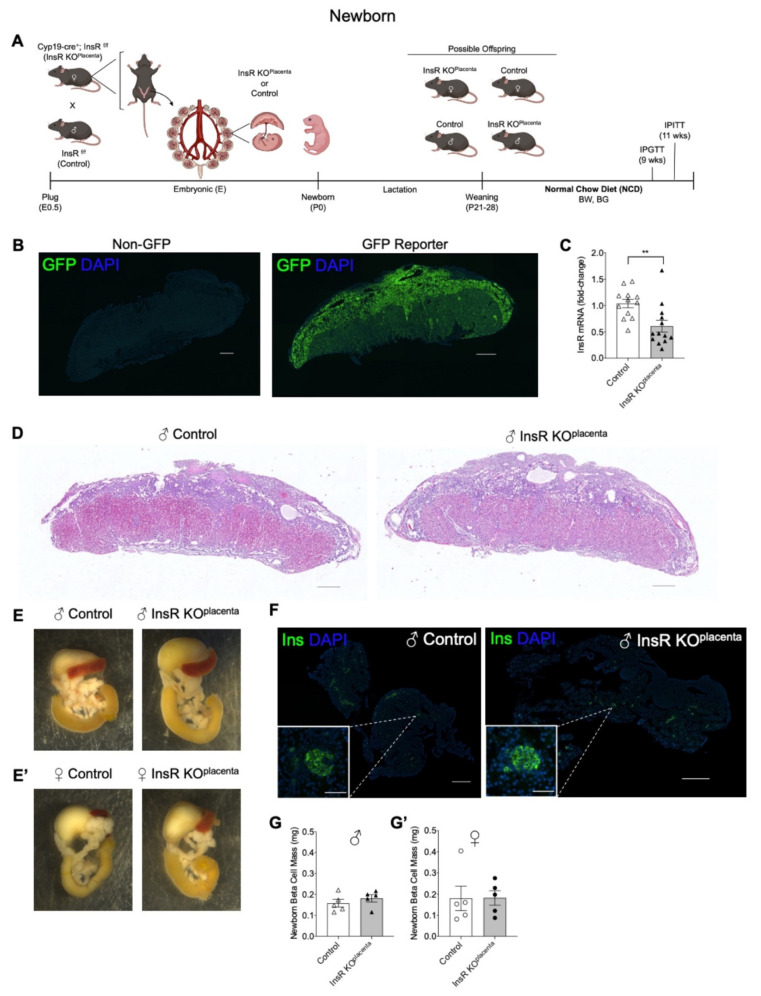
Newborn offspring with placenta-specific InsR ablation presented with normal body weight and pancreatic β-cell mass. (**A**) Experimental schematic for generating InsR KO^placenta^ and littermate controls. (**B**) GFP reporter (Cyp19-cre; CAG^+/+^) expressing endogenous green fluorescence (GFP) in a mouse embryonic E17.5 placenta section compared to the non-GFP control (CAG^+/+^) (scale bars: 500 μm). (**C**) InsR mRNA expression in the E17.5 InsR KO^placenta^ compared to controls by quantitative reverse transcription polymerase chain reaction (qPCR) (*n* = 12, 13). (**D**) Histology of the E17.5 male control placentas (left) compared to InsR KO^placenta^ (right) (scale bars: 500 μm). Background of images was subtracted post-imaging. (**E**) Gross morphology of the male and (**E’**) female control (left) and the InsR KO^placenta^ (right) newborn pancreata. (**F**) Whole pancreatic sections (scale bars: 500 μm) of the male newborn control (left) and the InsR KO^placenta^ (right) mice immunostained for insulin-positive islets (green) and DAPI (blue). Single islet β-cells (scale bars: 50 μm) shown as insets. Single islet images were cropped post-imaging to highlight the single islet. (**G**) Beta cell mass for the male (*n* = 5) and (**G’**) female (*n* = 5) newborns. The values are reported as the means ± SEM, ** *p* <0.01. E: embryonic; P: postnatal; BW: body weight; BG: blood glucose; IPGTT: intraperitoneal glucose tolerance test; IPITT; intraperitoneal insulin tolerance test; NCD: normal chow diet.

Using the Cyp19-cre recombinase, InsR was deleted genetically specifically in the placental trophoblast cells to generate InsR KO^placenta^ offspring and their littermate InsR floxed (InsR^f/f^) controls, as presented in the timeline of our study in Figure 1A. The efficiency of using the Cyp19-cre promoter to target specific genes within the placental trophoblast cell lineage was previously validated within our laboratory as well as by others [23,37,40]. In this study, we show that a cre reporter transgene expressing the green fluorescence protein (GFP) is expressed in the placental trophoblast cells where the Cyp19-cre recombinase is expected to be active and not in the control placenta (Figure 1B).

Quantitative reverse transcription polymerase chain reaction (qPCR) was performed on embryonic (E) 17.5 placentas to demonstrate effective reduction of InsR mRNA levels in the InsR KO^placenta^ placentas compared to their controls (*p* = 0.0055, Figure 1C). To assess alteration of InsR levels in the offspring (non-placental tissue), we measured InsR mRNA in the liver and adipose tissues of the adult offspring. The levels of the InsR transcript were comparable between InsR KO^placenta^ and their controls (data not shown), suggesting a placental specificity of Cyp19-cre in the placenta.

Placentas harvested on E17.5 did not differ in total weight between the InsR KO^placenta^ and their littermate controls (Figure S1A). Preliminary assessment of the gross placental morphology at E17.5 appears to be comparable between the littermate controls and the InsR KO^placenta^ placenta (Figure 1D), suggesting that placental IR is not required for the development of the placenta. Fetal body weight and pancreas weight at E17.5 also revealed no differences between the two genotypes (Figure S1B,C). The male and female InsR KO^placenta^ newborns presented with normal body weight compared to their respective controls (Table 1). There were also no differences in non-fasting blood glucose levels or serum insulin levels at birth among the groups (Table 1). Examination of the gross pancreas morphology, pancreas weight, and liver weight showed no apparent differences between genotypes for either male or female mice (Figure 1E, Table 1). Assessment of the basal pancreatic β-cell mass at birth demonstrated no differences between the InsR KO^placenta^ and their controls (Figure 1F,G,G’).

### 3.2. Adult InsR KO^placenta^ Mice Displayed Normal Glucose Homeostasis on a Normal Chow Diet

**Figure 2:**
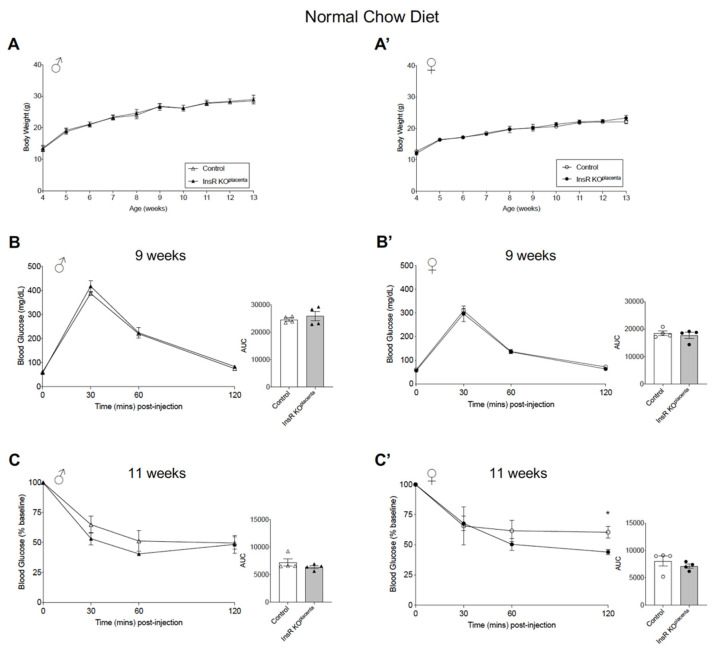
Adult InsR KO^placenta^ mice displayed normal glucose homeostasis on a normal chow diet. (**A**) Body weight in all the males (*n* = 9, 10) and (**A’**) the female (*n* = 13) InsR KO^placenta^ and control mice on a normal chow diet. Body weight for the offspring presented as combined data from parity ≥ 3 and ≤2 litters. (**B**) IPGTT and AUC analysis (right) for the male (*n* = 4) and (**B’**) female (*n* = 4) mice from parity ≥ 3 on a normal chow diet at 9 weeks of age. (**C**) IPITT and AUC analysis (right) for the males (*n* = 4) and (**C’**) females (*n* = 4) from parity ≥ 3 at 11 weeks of age. Blood glucose values for IPITT are expressed as the baseline percentage of blood glucose. The values are reported as the means ± SEM, * *p* < 0.05.

The InsR KO^placenta^ mice and their littermate controls were weaned onto a normal chow diet (NCD) at 4 weeks of age. There were no differences in the post-weaning body weight, measured from 4 to 13 weeks of age in male and female offspring, independent of parity (defined as the number of pregnancies the dam carried) (Figure 2A,A’). Fasting and non-fasting blood glucose levels, measured at 9 and 10 weeks of age, respectively, demonstrated no differences in either male or female mice from multiparous dams, which we defined as parity ≥ three pregnancies (Table 1). Glucose homeostasis in the adult offspring was assessed via an intraperitoneal (IP) glucose tolerance test (GTT) and an insulin tolerance test (ITT) performed at 9 weeks and 11 weeks of age, respectively. The male InsR KO^placenta^ mice from multiparous dams exhibited normal glucose and insulin tolerance (Figure 2B,C). The female InsR KO^placenta^ mice similarly demonstrated normal glucose tolerance but did show a significant change during the IPITT at timepoint T = 120 min (*p* = 0.0231) (Figure 2B’,C’). Analysis of the overall response (area under the curve, AUC), however, showed no significant differences in the female InsR KO^placenta^ mice compared to their controls (Figure 2C’). To assess whether the genotype of the dam (InsR^f/f^ vs. Cyp19-Cre; InsR^f/f^) impacts the glucose homeostasis of the adult offspring, glucose homeostasis measurements were performed on the InsR KO^placenta^ and control mice with either the dam or the sire as the carrier of the Cyp19-cre recombinase. Glucose and insulin tolerance remained unchanged (Figure S2). IPGTT and IPITT were performed on a separate cohort of offspring from parity ≤ 2, similarly demonstrating normal glucose and insulin tolerance (Figure S3A,A’,B,B’). Using a subset of mice from this group, IPGTT was performed at 20 weeks of age, which revealed that glucose tolerance did not differ between the two genotypes in either sex with age (Figure S3C,C’). We also generated heterozygous InsR Het^placenta^ (Cyp19-Cre; InsR^f/+^) and a respective control (InsR^f/+^) to assess a dosage effect of the InsR gene. There were no differences in glucose tolerance between the two genotypes in males and females (Figure S4).

### 3.3. Adult Male InsR KO^placenta^ Offspring Demonstrated a Lower Body Weight on a High-Fat Diet Challenge

**Figure 3:**
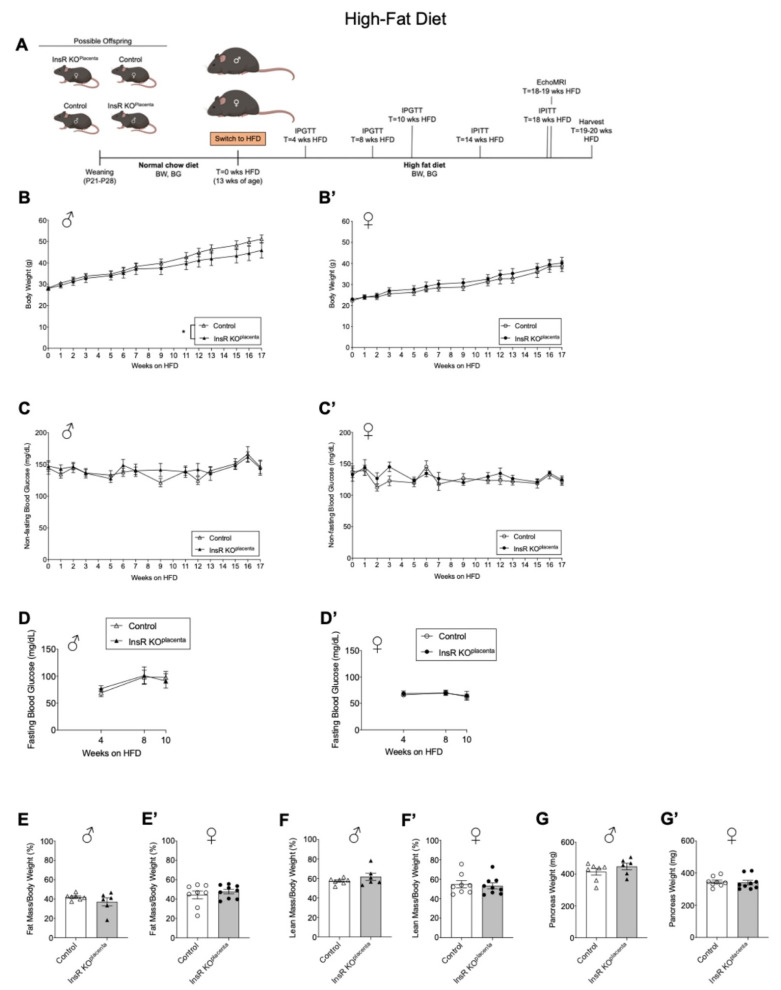
Adult male InsR KO^placenta^ offspring demonstrated lower body weight under a high-fat diet challenge. (**A**) Schematic of experimental design for a high-fat diet (HFD). (**B**) Body weight monitored in all the males (*n* = 6, 7) and (**B’**) females (*n* = 8, 9) from parity ≥ 3 and ≤2 across 17 weeks on a HFD. (**C**) Non-fasting blood glucose levels for all the males (*n* = 6, 7) and (**C’**) females (*n* = 8, 9) from parity was ≥3 and ≤2 (measured across 17 weeks on a HFD). Measurements not included for T = 4, 8, 10, 14, and 18 weeks on a HFD due to phenotyping performed on these weeks. (**D**) Fasting blood glucose measured for the males (*n* = 4, 7) and (**D’**) females (*n* = 4, 9) from parity ≥ 3 and ≤2 at 4, 8, and 10 weeks on a HFD. (**E**) Fat mass for the males (*n* = 6, 7) and (**E’**) females (*n* = 8, 9) from parity ≥ 3 and ≤2 as assessed by EchoMRI at 18–19 weeks on a HFD. (**F**) Lean mass for the males (*n* = 6, 7) and (**F’**) females (*n* = 8, 9) from parity ≥ 3 and ≤2 as assessed by EchoMRI at 18–19 weeks on a HFD. (**G**) Pancreas weight for the males (*n* = 6, 7) and (**G’**) females (*n* = 8, 9) from parity ≥ 3 and ≤2 upon harvest at 19–20 weeks on a HFD. The values are reported as the means ± SEM, * *p* < 0.05. P: postnatal; BW: body weight; BG: blood glucose; IPGTT: intraperitoneal glucose tolerance test; IPITT; intraperitoneal insulin tolerance test; HFD: high-fat diet.

Since we observed normal glucose tolerance under an NCD, we then tested whether placental InsR reduction increased the risk of obesity and metabolic dysfunction in the offspring under a diet-induced obesogenic challenge. Beginning at 13 weeks of age, the male and female InsR KO^placenta^ mice and their littermate controls were challenged with a high-fat diet (HFD, 60% energy by fat) to examine their response to an obesogenic challenge (Figure 3A). As expected, both the male and female mice, regardless of the genotype, increased in body weight over the 17 weeks on HFD treatment (Figure 3B,B’). Interestingly, when all the data from parity 1–4 were combined, the male InsR KO^placenta^ mice exhibited a significantly reduced body weight gain compared to their controls (*p* = 0.0112, Figure 3B). However, when parity ≤ 2 and ≥3 were considered separately, there were no differences (Figures S5A and S6A). In contrast, the female InsR KO^placenta^ mice did not differ in body weight compared to their controls over the 17 weeks on a HFD regardless of parity (Figure 3B’, Figures S5A’ and S6A’). Weekly non-fasted blood glucose measurements showed no notable differences between the InsR KO^placenta^ mice and their controls for either sex (Figure 3C,C’, Figures S5B,B’ and S6B,B’). Fasting blood glucose levels were measured after a 14 h fast at 4, 8, and 10 weeks on a HFD. No differences were observed in fasting blood glucose levels between the genotypes at any point in either sex regardless of parity (Figure 3D,D’). Body composition assessed by EchoMRI revealed no differences in fat mass or lean mass for either male or female mice after 18–19 weeks on a HFD (Figure 3E,E’,F,F’). Pancreas tissue harvested after 19–20 weeks on a HFD did not show weight differences between the genotypes for either the male or female mice regardless of parity (Figure 3G,G’). 

### 3.4. Adult Male InsR KO^placenta^ Offspring from Multiparous Dams Presented with a Mild and Transient Improved Glucose Homeostasis on a High-Fat Diet Challenge

**Figure 4:**
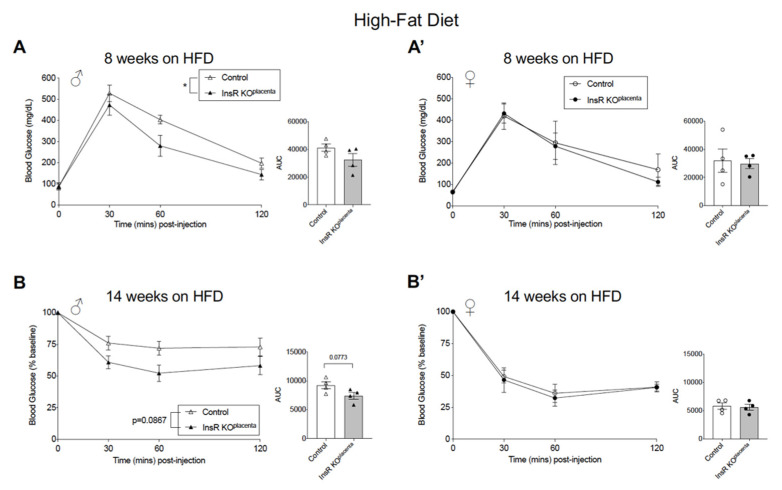
Adult male InsR KO^placenta^ mice from multiparous dams presented with a mild and transient improved glucose homeostasis on a high-fat diet challenge. (**A**) IPGTT and AUC analysis (right) for the male (*n* = 4) and (**A’**) female (*n* = 4) mice from parity ≥ 3 at 8 weeks on a HFD. Repeated measures two-way ANOVA revealed significant differences (*p* = 0.0332) between the InsR KO^placenta^ and control males at 8 weeks on a HFD. A separate unpaired *t*-test performed specifically for T = 60 min revealed *p* = 0.0593. (**B**) IPITT and AUC analysis (right) for the males (*n* = 4) and (**B’**) females (*n* = 4) from parity ≥ 3 at 14 weeks on a HFD. Blood glucose values for IPITT are expressed as the baseline percentage of blood glucose. Repeated measures two-way ANOVA analysis revealed a *p*-value of 0.0867 in the males at 14 weeks on a HFD. A separate unpaired *t*-test performed specifically for T = 30 and T = 60 min revealed *p* = 0.0843 and *p* = 0.0600, respectively. The values are reported as the means ± SEM, * *p* < 0.05.

In the male InsR KO^placenta^ mice on a HFD, the mild reduction in weight gain suggested a potential effect of reduced placental InsR on glucose homeostasis. IPGTT was conducted on the male and female InsR KO^placenta^ mice and their controls from either parity ≥ 3 or ≤2 at 4, 8, and 10 weeks on a HFD. At 4 weeks of a HFD, IPGTT results demonstrated no differences in glucose tolerance in either the male or female groups (Figure S5C,C’). Interestingly, at 8 weeks on a HFD, the male InsR KO^placenta^ mice from parity ≥ 3 demonstrated significantly improved glucose tolerance compared to their controls (*p* = 0.0332, Figure 4A). In contrast, there were no differences in the female group from parity ≥ 3 (Figure 4A’). IPGTT performed at 10 weeks on a HFD in the same group demonstrated a sustained improvement in glucose tolerance in the male InsR KO^placenta^ mice, but the effect was mild and did not reach statistical significance (*p* = 0.0899, Figure S5D). Glucose tolerance in the female group showed no difference at this time (Figure S5D’). Consistent with the mild improvement in glucose tolerance, IPITT conducted at 14 weeks on a HFD revealed a mild improvement in insulin tolerance in the male InsR KO^placenta^ mice from parity ≥ 3 compared to their controls (*p* = 0.0867, Figure 4B). There were no changes in insulin tolerance in the female group (Figure 4B’). IPITT was repeated at 18 weeks on a HFD to see if the improvement was sustained in the male InsR KO^placenta^ mice, but the results revealed no differences in insulin tolerance in the male or female mice at this time (Figure S5E,E’). IPGTT and IPITT were performed on a separate group of mice on a HFD from parity ≤ 2, but interestingly, the transient improvement in glucose homeostasis was not observed in this group (Figure S6C,C’,D,D’,E,E’). Within a subset of males from this group, activity levels and energy utilization were assessed using indirect calorimetry over a 48 h period. Both O_2_ consumption (day *p* = 0.0213; night *p* = 0.0272) and CO_2_ (day *p* = 0.0142; night *p* = 0.0165) production were significantly reduced in the InsR KO^placenta^ mice compared to the controls (Figure S7A,B). However, the respiratory exchange ratio (RER) and energy expenditure were comparable between the two groups (Figure S7C,D). Activity levels from day and night were significantly different within each genotype in the males (control *p* = 0.0003; InsR KO^placenta^ *p* = 0.0005), but between the two groups, the activity level was comparable (Figure S7E).

## 4. Discussion

Maternal obesity and diabetes during pregnancy continue to be serious public health concerns and are associated with adverse pregnancy complications for both the mother and the child [5,6,7,8,9,10,11,12,13,14,15]. In our present study, we aimed to understand the effect of altered insulin receptor availability in the placenta on the metabolic health of the mouse offspring. We uncovered that placenta-specific insulin receptor deficiency in a normal mouse pregnancy condition does not adversely affect glucose homeostasis in the male or female offspring at birth and during adulthood. Under a diet-induced obesogenic challenge, however, adult male offspring with a placental insulin receptor deletion displayed transient protection from glucose homeostasis dysfunction, which appears to be dependent on the number of pregnancies the dam has experienced. 

Placental trophoblast-specific loss of the insulin receptor did not affect placental weight, fetal and newborn weight, or β-cell mass in the male or female newborns. These results were consistent with findings that placental trophoblast-specific deletion of InsR alone does not adversely impact offspring growth in mice [40]. Another study looking at the effects of a total body insulin receptor knockout also found normal fetal development with normal or slightly reduced body weight at birth [41,42]. The minimal impact on body weight is likely attributable to the effects of insulin-like growth factor 1 receptor (IGF1R) in normal fetal growth and development [41,42]. The insulin receptor and the IGF1 receptor (IGF1R) share high homology in their ligand-binding and intracellular tyrosine kinase domains, and thus insulin can activate both receptors (not IGF2R) albeit with lower affinity to IGF1R [43,44,45]. Thus, future studies into the independent role of IGF1R and the combined role of InsR and IGF1R in the placenta may reveal new insights regarding the full effect of placental insulin signaling, which will better inform us of the full effects of maternal insulin on birthweight and β-cell mass at birth.

Glucose and insulin tolerances of the adult InsR KO^placenta^ offspring on a normal chow diet were unaffected and suggest that placental InsR on its own is not sufficient to induce lasting effects on glucose homeostasis of the offspring. Normal glucose tolerance in the adult offspring was also observed by Bronson et al. [40]. However, when challenged with a HFD, male InsR KO^placenta^ mice demonstrated lower body weight gain compared to their littermate controls. Male InsR KO^placenta^ offspring also demonstrated improved glucose and insulin tolerance, although this was a mild and transient effect dependent on maternal parity. The improved glucose and insulin tolerance was demonstrated in the cohort from multiparous dams, which we defined as having ≥ three pregnancies. We observed no changes in glucose homeostasis parameters in different cohorts of offspring from dams with parity ≤ 2. While we are careful in the interpretation of this analysis, deletion of the placental insulin receptor may potentially be associated with a mild protective effect against diet-induced obesity and glucose homeostasis dysfunction in male InsR KO^placenta^ offspring when challenged with a HFD. This phenotype appears to be dependent on the number of pregnancies the dam has experienced. Thus, ablation of the placental InsR may have health beneficial effects in the male offspring of multiparous dams, where they often present higher obesity [46,47] and hyperinsulinemia [48]. Indeed, epidemiological and experimental studies have shown that increased parity may be associated with an increased risk of maternal diabetes, glucose homeostasis dysfunction, and placental inflammation [17,33,49,50]. In a preclinical model, there is also evidence demonstrating that male offspring from multiparous dams have increased adiposity and metabolic dysfunction [17]. We are uncertain what factors may be mediating this effect in multiparous dams, but repeated pregnancies may adversely change the intrauterine environment through changes related to pregnancy-induced obesity, maternal and placental inflammation, maternal hyperinsulinemia, and placental nutrient transport [17]. Interestingly, placental insulin receptor ablation was also shown to significantly increase hypothalamic-pituitary–adrenal axis stress response and impair sensorimotor gating in males [40]. Thus, sex-specific neurodevelopmental and metabolic risk programming should be investigated in the future.

## 5. Conclusions and Future Directions

In the present study, we explored the role of the placental insulin receptor in a normal mouse pregnancy using a preclinical model, and our results suggest that placental InsR may contribute to the long-term offspring health when metabolically challenged with diet-induced obesity. This study suggests that reduction of placental insulin in dams with hyper-nutrient conditions such as obesity and hyperinsulinemia (i.e., PCOS) may improve metabolic health of the offspring. Our data highlights the need for a greater understanding of the role of insulin signaling in placental biology and the mechanisms of developmental programming of the metabolic health in the offspring. This study also highlights that multiple pregnancies may have heritable and independent consequences in the offspring and warrants further investigation using preclinical models and in human studies.

Future studies should be directed toward exploring more closely the impact of parity on the metabolic health of the dams and the offspring by characterizing glucose homeostasis of multiparous wild type or InsR KO dams to define how their metabolic health may be altered with an increasing number of pregnancies. With a more robust amount of dams, confounding variables such as the dams’ age, weight gain between pregnancies, and litter effects within dams corrected, a more thorough study may shed some insight into why male offspring with a placental deletion of the insulin receptor may present with a mildly protective phenotype. Assessment of the offspring’s metabolic health if the sire is a Cre carrier should also be considered. Fetal sex has been shown to play a role in sex-specific responses to changes in the in utero environment and may partially explain the sexual dimorphic differences observed in our study and others [23,40,51,52,53]. Thus, an important future study should also investigate the role of the insulin receptor in placental nutrient-sensing and placental vascularization. Moreover, future interventions may be targeted to break the obesity cycle that could occur between mothers and their offspring. 

## Figures and Tables

**Table 1:** Male and female offspring characteristics in newborns (P0) and adults (parity ≥ 3) on a normal chow diet. Newborn characteristics in terms of body weight, non-fasting blood glucose, non-fasting serum insulin, pancreas weight, and liver weight. Adult offspring characteristics in terms of fasting and non-fasting blood glucose levels measured at 9 and 10 weeks of age, respectively. The data are presented as the means ± SEM.

	Male Offepring		Female Offspring	
Newborn (P0)	Control	InsR KO^placenta^	Control	InsR KO^placenta^
Body weight	1.226 ± 0.03039	1.318 ± 0.03808	1.242 ± 0.02603	1.251 ± 0.03923
Non-fasting blood glucose	39.67 ± 6.888	36.50 ± 3.833	33.17 ± 5.183	34.90 ± 3.093
Non-fasting serum insulin	0.3919 ± 0.1794	0.4409 ± 0.1791	0.4676 ± 0.1500	0.4622 ± 0.1129
Pancreas weight	7.514 ± 0.3269	8.079 ± 0.4086	8.093 ± 0.6240	8.207 ± 0.6032
Liver weight	46.33 ± 3.449	49.26 ± 2.904	47.92 ± 2.351	49.58 ± 3.411
**Adult (Parity ≥ 3) Normal Chow Diet**	**Control**	**InsR KO^placenta^**	**Control**	**InsR KO^placenta^**
Fasting blood glucose (9 weeks old)	61.25 ± 1.652	57.25 ± 4.029	60.25 ± 2.175	56.25 ± 2.810
Non-fasting blood glucose (10 weeks old)	132.5 ± 13.85	135.0 ± 5.212	138.3 ± 18.03	126.8 ± 13.81

## Data Availability

Not applicable.

## Supplementary Materials

The following supporting information can be downloaded at: https://www.mdpi.com/article/10.3390/biomedicines10030575/s1, Figure S1. Normal placental and fetal weight in InsR KOplacenta offspring. Figure S2. Normal glucose homeostasis in InsR KOplacenta offspring from sire Cyp19-cre carrier. Figure S3. A separate cohort of adult InsR KOplacenta mice from parity ≤ 2 demonstrated normal glucose homeostasis on normal chow diet. Figure S4. Placental InsR heterozygous mice demonstrated normal glucose homeostasis on normal chow diet. Figure S5. Adult male InsR KOplacenta mice from multiparous dams presented with a mild and transient improved glucose homeostasis on a high-fat diet challenge. Figure S6. Adult male InsR KOplacenta mice from parity ≤ 2 exhibited normal glucose homeostasis on a high-fat diet challenge. Figure S7. Adult male InsR KOplacenta mice displayed normal energy expenditure on a high-fat diet challenge.
