# Supplementary material for: Placental Insulin Receptor Transiently Regulates Glucose Homeostasis in the Adult Mouse Offspring of Multiparous Dams
Source: Biomedicines. 2022 Mar 1;10(3):575. doi: 10.3390/biomedicines10030575 (PMC8945682; doi:10.3390/biomedicines10030575)
Supplement: Supplementary file 1 [file biomedicines-10-00575-s001.zip › biomedicines-1603817-supplementary.pdf]

Figure S1

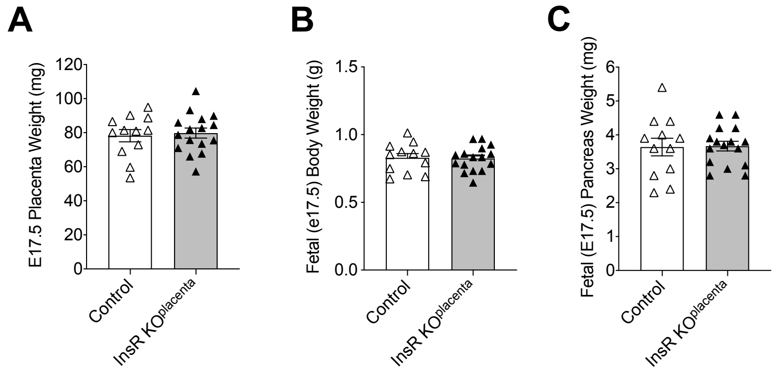

A
B
C
E17.5 Placenta Weight (mg)
120
100
80
60
40
20
0
Control
InsR KOplacenta
Fetal (e17.5) Body Weight (g)
1.5
1.0
0.5
0.0
Control
InsR KOplacenta
Fetal (E17.5) Pancreas Weight (mg)
6
5
4
3
2
1
0
Control
InsR KOplacenta

Figure S2

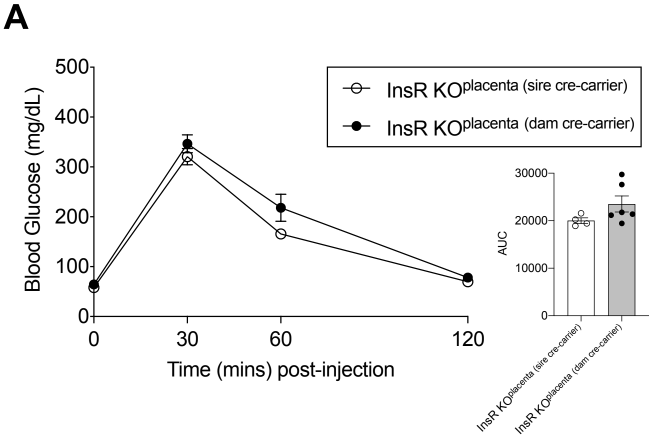
A
Blood Glucose (mg/dL)
500
400
300
200
100
0
0
30
60
120
Time (mins) post-injection
InsR KOplacenta (sire cre-carrier)
InsR KOplacenta (dam cre-carrier)
AUC
30000
20000
10000
0

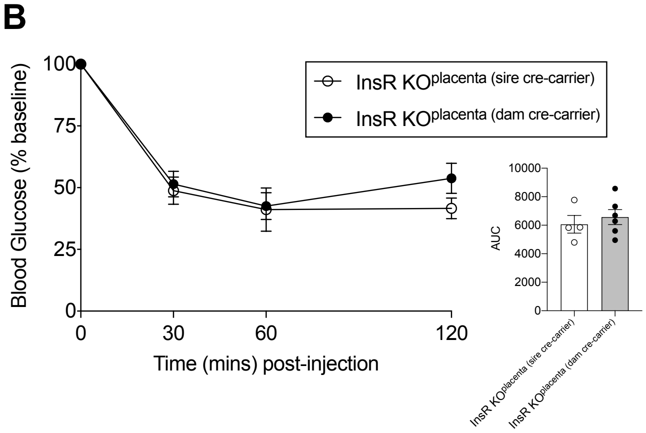
B
Blood Glucose (% baseline)
100
75
50
25
0
0
30
60
120
Time (mins) post-injection
InsR KOplacenta (sire cre-carrier)
InsR KOplacenta (dam cre-carrier)
AUC
10000
8000
6000
4000
2000
0

Figure S3 Normal Chow Diet, Parity ≤ 2

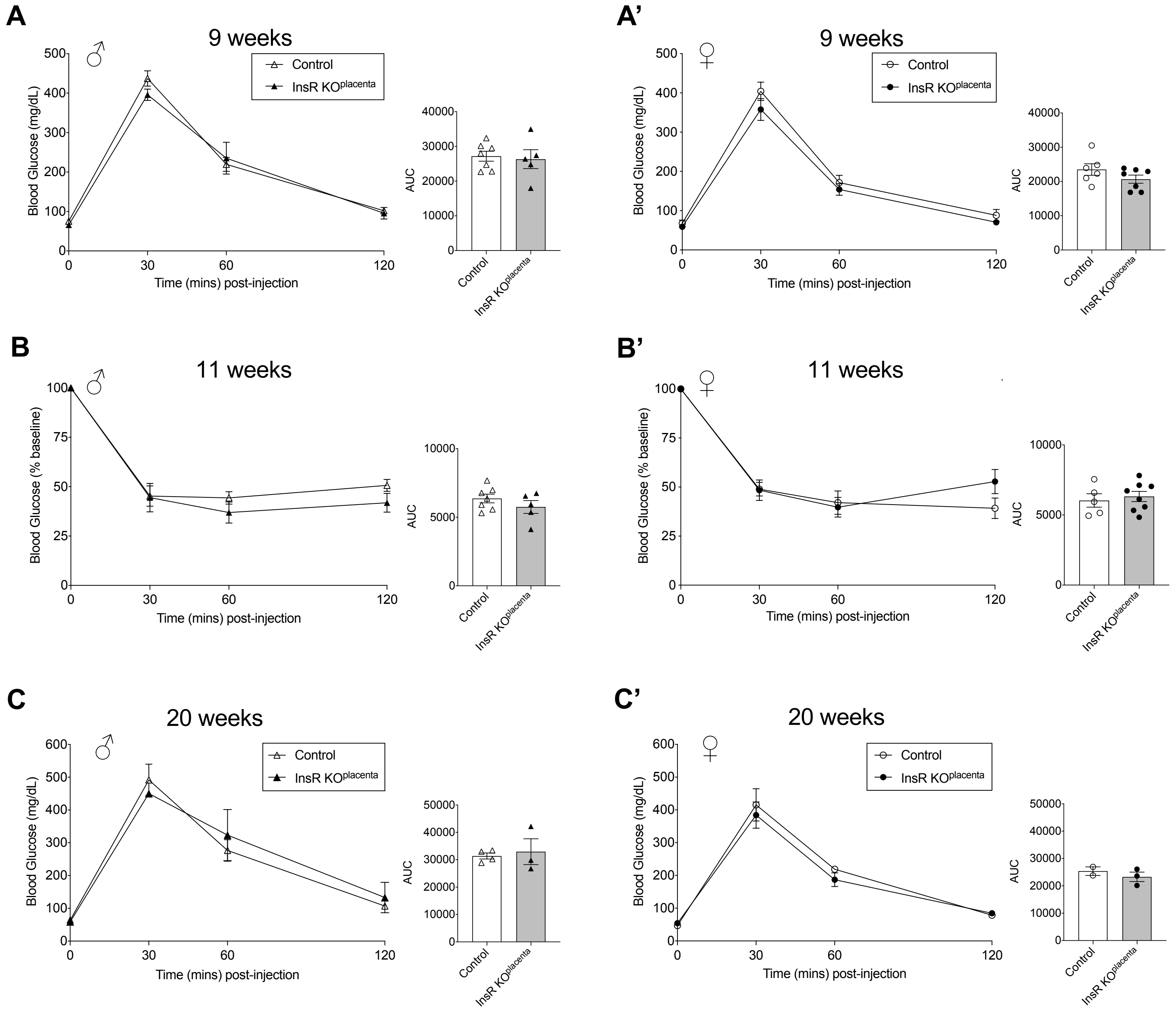

Figure S4

Heterozygous $InsR^{f/+}$

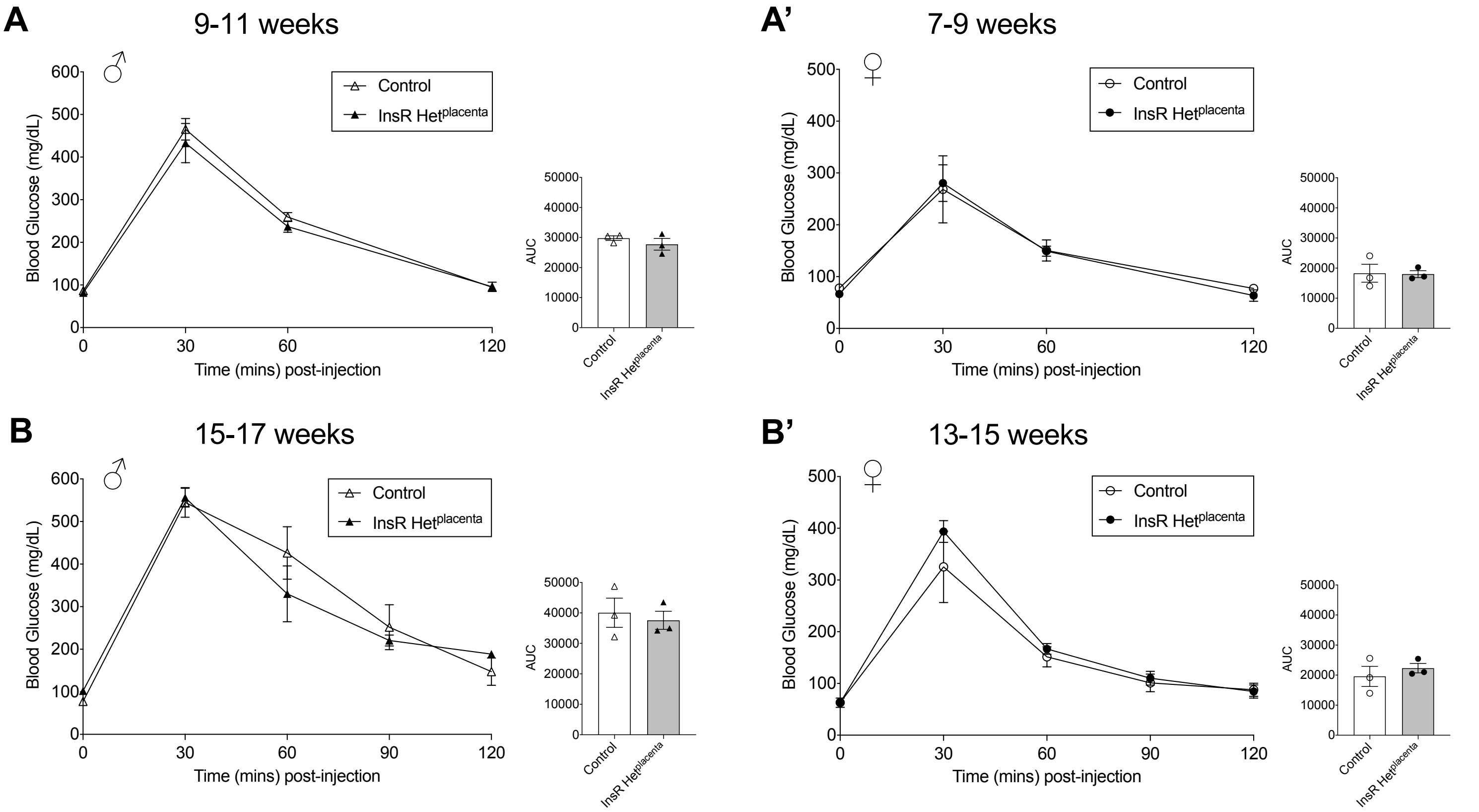

Figure S5

High-Fat Diet, Parity ≥ 3

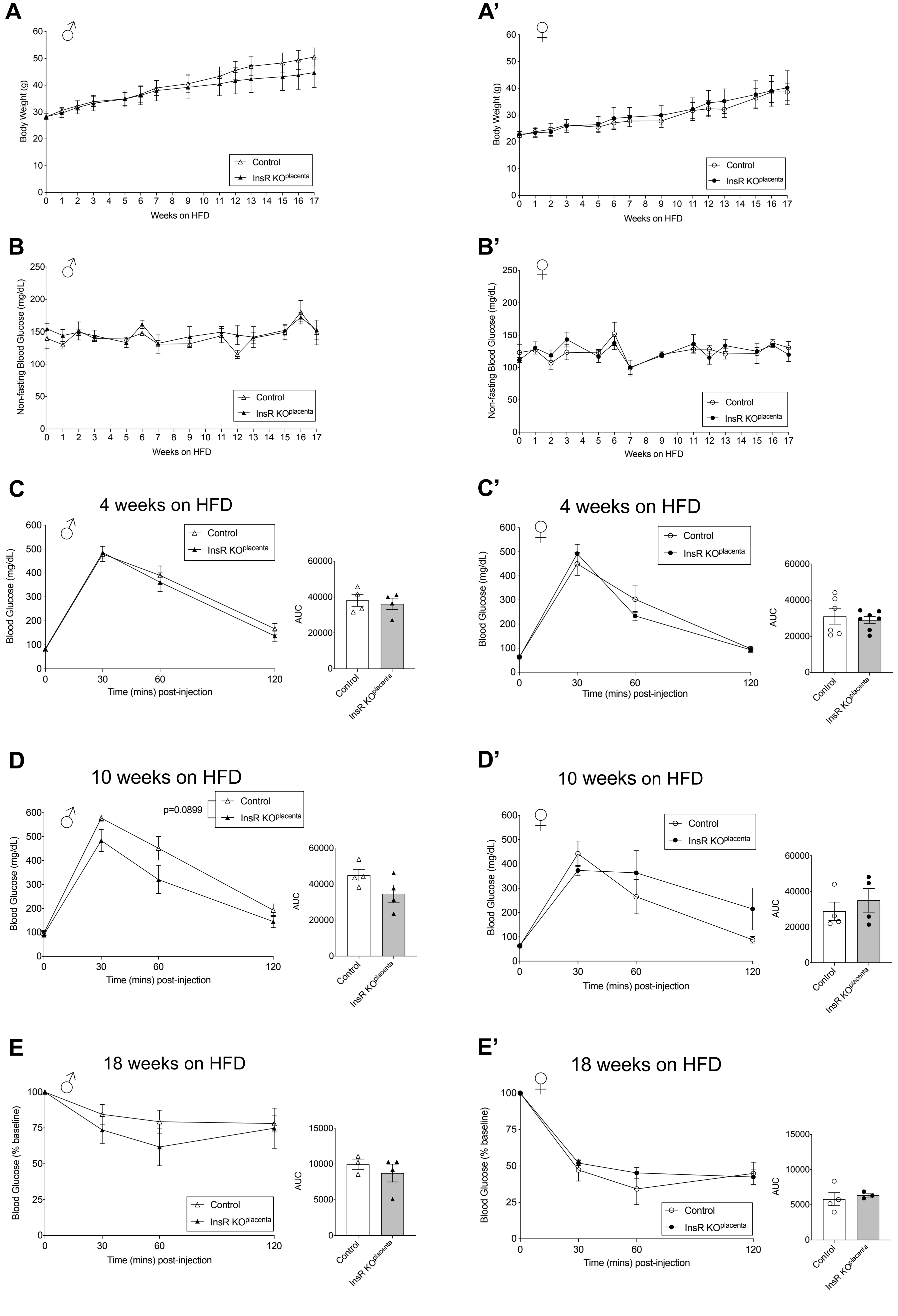

Figure S6

High-Fat Diet, Parity ≤ 2

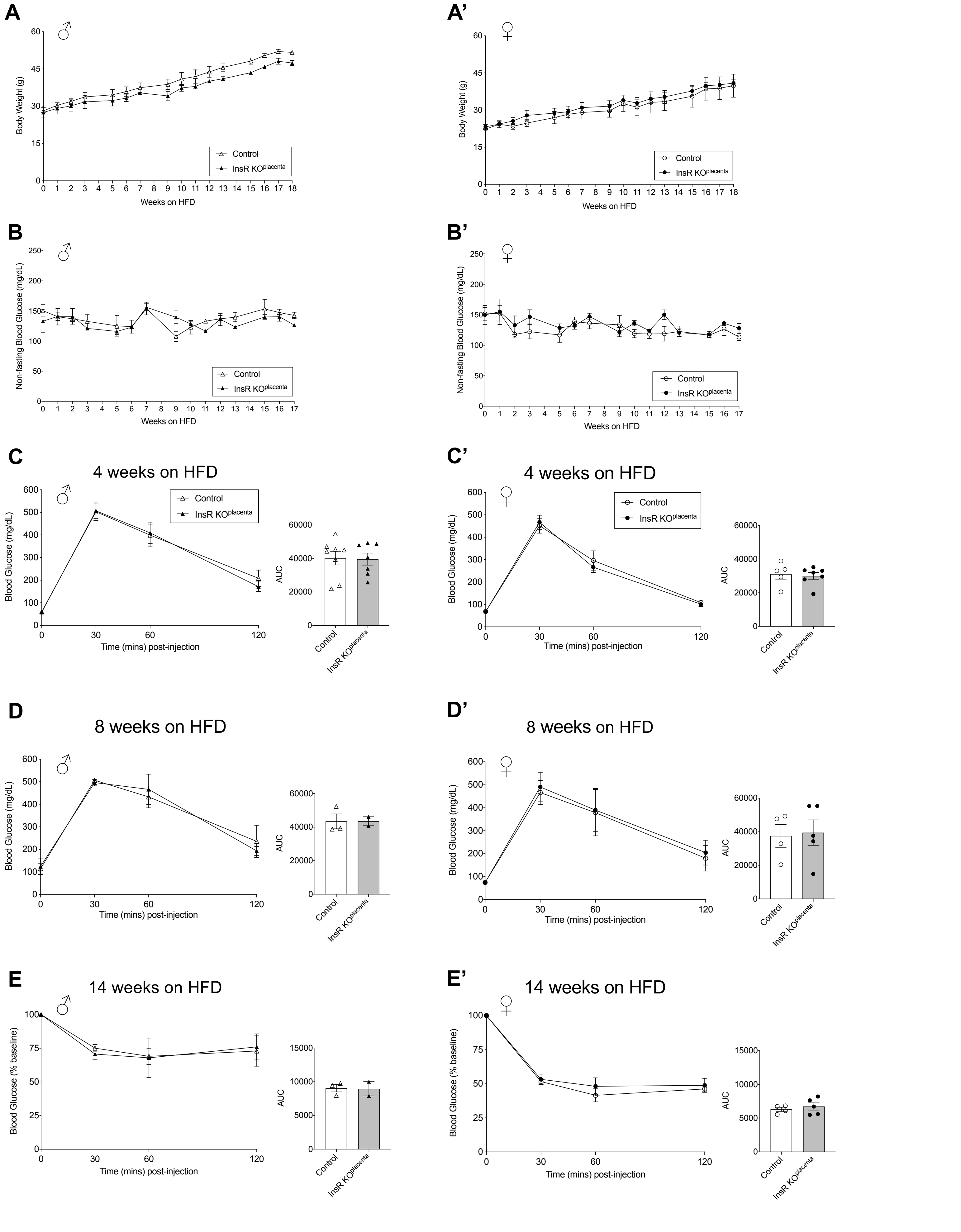

Figure S7

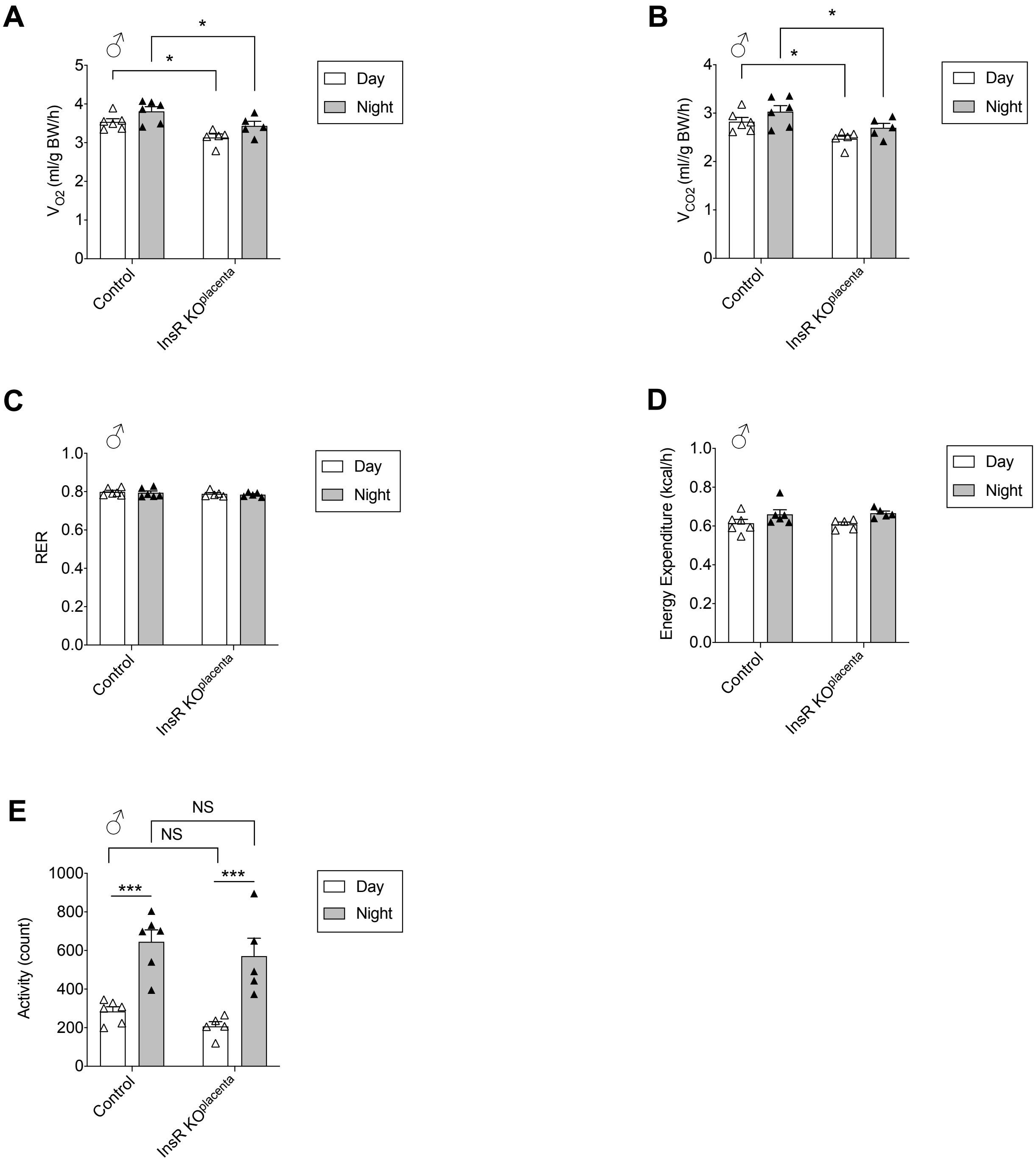

A
♂
*
*
V_{O2} (ml/g BW/h)
0
1
2
3
4
5
Day
Night
Control
InsR KO^placenta
B
♂
*
*
V_{CO2} (ml/g BW/h)
0
1
2
3
4
Day
Night
Control
InsR KO^placenta
C
♂
RER
0.0
0.2
0.4
0.6
0.8
1.0
Day
Night
Control
InsR KO^placenta
D
♂
Energy Expenditure (kcal/h)
0.0
0.2
0.4
0.6
0.8
1.0
Day
Night
Control
InsR KO^placenta
E
♂
NS
NS
***
***
Activity (count)
0
200
400
600
800
1000
Day
Night
Control
InsR KO^placenta

Supplemental Figure Legends:

Figure S1. Normal placental and fetal weight in InsR KO^{placenta} offspring.
(A) Embryonic (E) 17.5 placental weight (n = 7, 10). **(B)** Fetal (E17.5) weight (n = 7, 10). **(C)** Fetal E17.5 pancreas weight (n = 7, 10). Values reported as mean ± SEM, *$p < 0.05$.

Figure S2. Normal glucose homeostasis in InsR KO^{placenta} offspring from sire Cyp19-cre carrier. (A) IPGTT and AUC analysis (right) for offspring on normal chow diet at 9 weeks of age (n = 4, 6). **(B)** IPITT and AUC analysis (right) for offspring at 11 weeks of age (n = 4, 6). Blood glucose values for IPITT are expressed as a percent baseline of blood glucose. Values reported as mean ± SEM, *$p<0.05$.

Figure S3. A separate cohort of adult InsR KO^{placenta} mice from parity ≤ 2 demonstrated normal glucose homeostasis on normal chow diet. (A) IPGTT and AUC analysis (right) for male (n = 5, 7) and **(A′)** female (n = 6, 7) mice from parity ≤ 2 on normal chow diet at 9 weeks of age. **(B)** IPITT and AUC analysis (right) for males (n = 5, 7) and **(B′)** females (n = 6, 7) from parity ≤ 2 at 11 weeks of age. Blood glucose values for IPITT are expressed as a percent baseline of blood glucose. **(C)** IPGTT and AUC analysis (right) for male (n = 3, 4) and **(C′)** female (n = 2, 3) mice on normal chow diet at 20 weeks of age. Values reported as mean ± SEM, *$p<0.05$.

Figure S4. Placental InsR heterozygous mice demonstrated normal glucose homeostasis on normal chow diet. (A, B) IPGTT and AUC analysis for male (n = 3) and **(A′, B′)** female (n = 3) adult mice on normal chow. Values reported as mean ± SEM, *$p<0.05$.

Figure S5. Adult male InsR KO^{placenta} mice from multiparous dams presented with a mild and transient improved glucose homeostasis on a high-fat diet challenge. (A) Body weight monitored in males (n = 4) and **(A′)** females (n = 4) from parity ≥ 3 across 17 weeks on HFD. **(B)** Non-fasting blood glucose levels for males (n = 4) and **(B′)** females (n = 4) from parity ≥ 3 measured across 17 weeks on HFD. **(C)** IPGTT and AUC analysis (right) for male (n = 4) and **(C′)** female mice (n = 6, 7) from parity ≥ 3 at 4 weeks on HFD. **(D)** IPGTT and AUC analysis (right) for male (n = 4) and **(D′)** female mice (n = 4) from parity ≥ 3 at 10 weeks on HFD. Two-way ANOVA did not reveal significance at specific time points in the males. Unpaired *t*-test done specifically for T=30 minutes revealed p=0.0951. **(E)** IPITT and AUC analysis (right) for males (n = 4) and **(E′)** females (n = 3, 4) from parity ≥ 3 at 18 weeks on HFD. Blood glucose values for IPITT are expressed as a percent baseline of blood glucose. Values reported as mean ± SEM, *$p<0.05$.

Figure S6. Adult male InsR KO^{placenta} mice from parity ≤ 2 exhibited normal glucose homeostasis on a high-fat diet challenge. (A) Body weight of males (n = 2, 3) and **(A′)** females (n = 4, 5) from parity ≤ 2 across 18 weeks on HFD. Body weight not included for T = 4, 8, and 14 weeks on HFD due to phenotyping performed on those weeks. **(B)** Non-fasting blood glucose levels for males (n = 2, 3) and **(B′)** females (n = 4, 5) across 17 weeks on HFD. **(C)** IPGTT and AUC analysis (right) for male (n = 7, 8) and **(C′)** female mice (n = 5, 7) from parity ≤ 2 at 4 weeks on HFD. **(D)** IPGTT and AUC analysis (right) for male (n = 2, 3) and **(D′)** female mice (n = 4, 5) from parity ≤ 2 at 8 weeks on HFD. **(E)** IPITT and AUC analysis (right) for males (n = 2, 3) and **(E′)** females (n = 4) from parity ≤ 2 at 14 weeks on HFD. Blood glucose values for IPITT are expressed as a percent baseline of blood glucose. Values reported as mean ± SEM, *$p<0.05$.

Figure S7. Adult male InsR KO^{placenta} mice displayed normal energy expenditure on a high-fat diet challenge. (A) Volume of oxygen (VO_2) consumption and **(B)** volume of carbon dioxide (VCO_2) exchange ratio (RER) over 48 h (n = 5, 6) with 12 h light and dark cycles. Dark cycle indicated by shaded region. **(D)** Energy expenditure over 48 h (n = 5, 6) with 12 h light and dark cycles. Dark cycle indicated by shaded region. **(E)** Activity level measured by beam breaks (n = 5, 6). Values reported as mean ± SEM, *$p<0.05$.
